# Time From First Contact With the Heart Team to Transcatheter Aortic Valve Replacement (TAVR) and the Area Deprivation Index (ADI)

**DOI:** 10.7759/cureus.105110

**Published:** 2026-03-12

**Authors:** Caroline K Chen, Matthew J Billy, Christian Summa, Zachary Brennan, Harrison Dean, Tariq Ahmad, Tyler J Wallen

**Affiliations:** 1 General Surgery, Geisinger Health System, Scranton, USA; 2 General Surgery, Geisinger Health System, Wilkes-Barre, USA; 3 Cardiac Surgery, Smidt Heart Institute, Cedars-Sinai Medical Center, Los Angeles, USA; 4 Medicine, New York Institute of Technology College of Osteopathic Medicine, Jonesboro, USA; 5 Interventional Cardiology, Geisinger Wyoming Valley Medical Center, Wilkes-Barre, USA; 6 Cardiovascular Surgery, Geisinger Health System, Wilkes-Barre, USA

**Keywords:** area deprivation index, cardiothoracic surgery, socioeconomic status, surgical aortic valve repair, transcatheter aortic valve repair

## Abstract

Introduction

Transcatheter aortic valve replacement (TAVR) is an increasingly used form of aortic valve replacement. Prolonged wait time prior to TAVR is associated with an increased mortality rate. This study examines whether the extent of neighborhood disadvantage of patients has any impact on time to TAVR, and if so, to what degree.

Methods

After institutional review board approval, we conducted a retrospective review of a prospectively maintained database to assess time to TAVR. Time to TAVR was defined from first contact with the structural heart team until implant. Inclusion criteria involved patients undergoing TAVR from January 2019 until January 2024. The area deprivation index (ADI) was determined utilizing the patient’s listed home address and the Neighborhood Atlas developed by the Center for Health Disparities Research of the University of Wisconsin School of Medicine and Public Health.

Results

The patient population included 708 patients. No significant correlation between time to TAVR and ADI was found for patients with a time to TAVR one or two standard deviations from the mean. For patients with time to TAVR greater than three standard deviations from the mean, positive correlations were found between time to TAVR and ADI, as well as time to TAVR and national percentile. The national percentile was obtained by comparing ADI scores for the entire United States, which were then ranked and divided into percentiles.

Conclusions

Patients from the most disadvantaged neighborhoods experience a longer time to TAVR than others. We hypothesized that a low socioeconomic status would potentially cause barriers with follow-up; however the prolonged time to TAVR in this study was not shown to be secondary to that.

## Introduction

Transcatheter aortic valve replacement (TAVR) is an increasingly used form of aortic valve replacement (AVR), emerging as a preferred alternative to surgical aortic valve replacement (SAVR), particularly in patients considered at high or intermediate risk for open-heart procedures [1,2]. Landmark trials have demonstrated the safety and efficacy of TAVR in even low-surgical-risk populations [2,3]. As a result, the procedure has seen rapid global adoption and now constitutes the majority of AVRs in the United States [4]. As the demand for TAVR continues to grow, ensuring timely access to the procedure has become increasingly critical. Recent studies have shown that delays in time to TAVR are associated with adverse outcomes. Wijeysundera and colleagues demonstrated that a wait time of >60 days effectively eliminates TAVR’s survival advantage over SAVR [5]. This dynamic was examined in the context of the COVID-19 pandemic by comparing the time from first contact to TAVR across three pandemic waves relative to pre-COVID-19 data at an institution [6]. This study revealed a significant increase in mean time to TAVR during each wave, along with a statistically significant rise in all-cause postoperative mortality compared to the pre-pandemic period [6].

It is well-established that socioeconomic factors influence access to cardiovascular care [7]. These factors can include social determinants of health, such as socioeconomic status, education level, and neighborhood environment [7]. For example, among U.S. Medicare patients with severe aortic stenosis, those in the most deprived communities were significantly less likely to undergo any AVR procedure [8]. One metric that can be utilized to assess these factors on access to and timing of care is the area deprivation index (ADI). ADI is a composite measure of neighborhood disadvantage that quantifies socioeconomic conditions by incorporating 17 US Census indicators of income, education, employment, and housing quality [9]. Each geographic area, often a census block group, is assigned an ADI score and national percentile, with higher values indicating greater socioeconomic deprivation [8]. The ADI data is made public through the Neighborhood Atlas developed by the University of Wisconsin School of Medicine and Public Health, and this index has been widely used to study health disparities [10]. Prior research has linked higher ADI to worse health outcomes and access to care in multiple contexts, making it a useful tool for investigating the impact of neighborhood-level socioeconomic status on healthcare delivery [9,11].

National registry analyses have found that areas with higher deprivation indices exhibit a lower prevalence and slower adoption of TAVR over time​ [10]. To the best of our knowledge, our work is the first to study the impact of social deprivation on access to TAVR as measured by wait times [12]. The present study aims to evaluate whether the extent of neighborhood disadvantage of patients, measured by the ADI, has any impact on time to TAVR, and if so, to what degree. The strengths of our study were that we evaluated all patients on the TAVR waiting list and used a multidimensional index to measure social deprivation, along with its effects on patient outcomes.

This article was previously presented as a meeting abstract at the 2024 Eastern Cardiothoracic Surgical Society Conference on September 21, 2024.

## Materials and methods

Institutional review board approval was obtained following our institutional guidelines. This study is a retrospective review of a prospectively maintained database to assess time to TAVR. Patient data were obtained from two hospital facilities within a single institution, utilizing our institution’s Electronic Medical Record and our own prospectively maintained TAVR database. Time to TAVR was defined from first contact with the structural heart team until implant.

Inclusion criteria involved patients undergoing TAVR from January 2019 until January 2024. The patient population included 396 males and 310 females. ADI was determined utilizing the patient’s listed home address and the Neighborhood Atlas developed by the Center for Health Disparities Research of the University of Wisconsin School of Medicine and Public Health. Statistical analysis was performed using the Pearson correlation coefficient, examining the ADI and national percentile versus our time to TAVR, and plotted on a bar graph.

## Results

A total of 708 patients who underwent TAVR between January 2019 and January 2024 were included in the study. The cohort represented a broad geographic and socioeconomic range as defined by the ADI based on patient home address.

**Figure 1:**
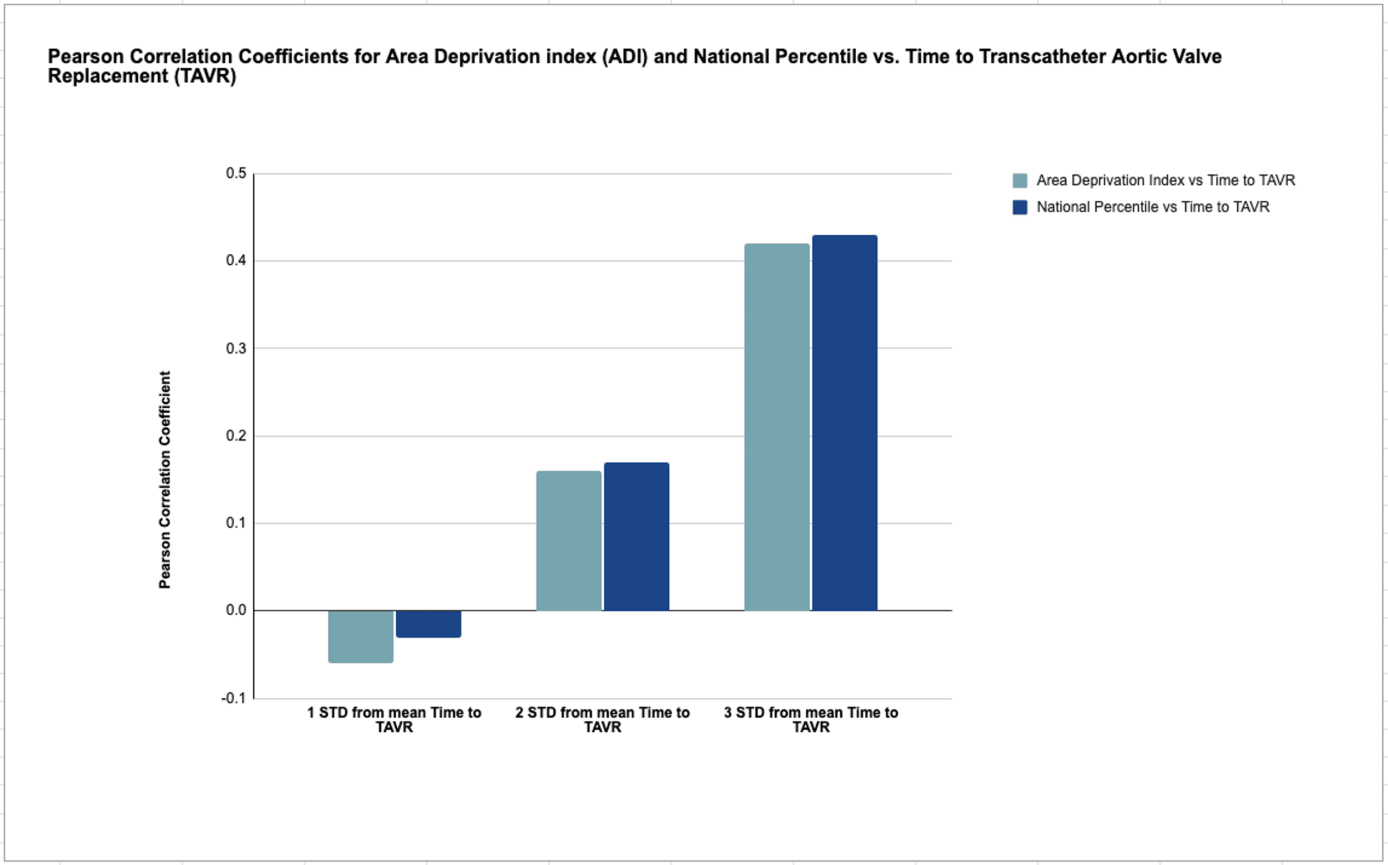
Pearson correlation coefficients for area deprivation index (ADI) and national percentile vs. time to transcatheter aortic valve replacement (TAVR) by standard deviations from the mean time to TAVR

The mean time from initial contact with the structural heart team to TAVR implant was calculated for the entire cohort. For the majority of patients-those whose time to TAVR fell within 1 or 2 standard deviations of the mean-no significant correlation was found between time to TAVR and ADI or national ADI percentile (p > 0.05 for both) (Figure 1).

**Figure 2:**
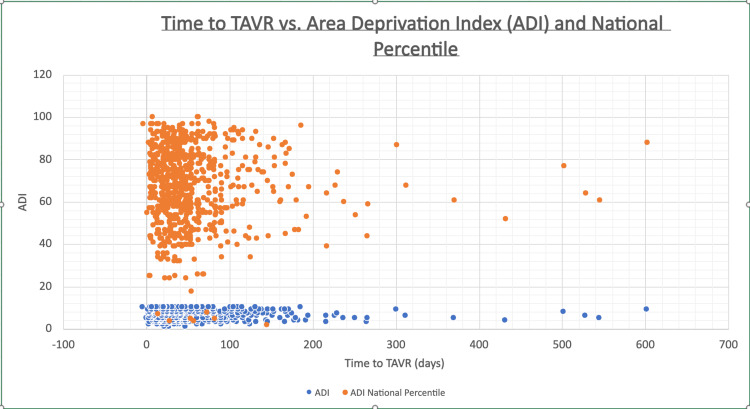
Correlation between time to TAVR and (A) area deprivation index (ADI) and (B) National ADI percentile

However, among the subset of patients with time to TAVR greater than three standard deviations above the mean, statistically significant positive correlations were observed. In this group, higher ADI scores and national percentile rankings were associated with longer time to TAVR. Specifically, Pearson correlation coefficients were r = 0.42 for ADI and r = 0.43 for national percentile (p < 0.05 for both), suggesting a moderate relationship between lower socioeconomic deprivation and increased procedural delay (Figure 2).

These findings indicate that while neighborhood-level socioeconomic disadvantage does not impact the majority of patients undergoing TAVR, there are significant associations for the most disadvantaged patients, patients with time to TAVR > 3 standard deviations from the mean. This may reflect compounding barriers to timely care in the most disadvantaged populations.

## Discussion

This study investigated whether neighborhood-level socioeconomic disadvantage, as measured by the ADI, influences time from first contact with the heart team to TAVR. ADI scores take into account the effect of physical, chemical, and social influences on human pathophysiology [8]. Neighborhood disadvantages are associated with higher rates of several diseases, including cardiovascular disease and dementia [7]. Part of the connection involves common risk factors for cardiovascular disease, such as hypertension, hyperlipidemia, and diabetes, which are seen at higher rates in lower socioeconomic status households [7].

While no significant correlation was found for the majority of patients, our results show that among those experiencing extreme delays (greater than three standard deviations from the mean time to TAVR), higher ADI scores and national percentiles were noted. These findings suggest that socioeconomic deprivation may not broadly affect access for most patients, but it does appear to be a critical factor for a subset facing substantial delays.

This observation aligns with prior research showing that patients from disadvantaged neighborhoods are less likely to undergo TAVR or may face longer wait times for advanced cardiovascular interventions [7,10]. However, our study is among the first to isolate the influence of ADI on time to TAVR after referral, rather than on access or procedural rates overall. This distinction is important; while systemic referral barriers may limit access in broader populations, our data suggests that even after referral, neighborhood-level disadvantage continues to affect care timelines, particularly at the extremes. It is not enough to simply gain access to care. There are socioeconomic effects on the entire process of continued patient care.

Several mechanisms may explain this delay. Patients from highly deprived areas may face more complex logistical barriers, including limited transportation, competing health or caregiving responsibilities, or difficulty navigating follow-up and testing schedules. While we hypothesized that follow-up noncompliance might contribute, our review found no consistent evidence to support this as the primary driver. This suggests the need for further research into unmeasured social determinants such as access to support systems, communication gaps, and institutional biases in follow-up management.

Interestingly, for patients within one or two standard deviations of the mean time to TAVR, ADI had no significant effect. This may reflect the effectiveness of structured heart team protocols and multidisciplinary coordination in standard cases, which could buffer against socioeconomic disparities. However, this protective effect seems to break down in outlier cases, raising questions about how well systems adapt to patients with more complex socioeconomic profiles.

Our findings emphasize the importance of identifying patients at risk for procedural delay early in the TAVR pathway. Incorporating ADI or similar metrics into preoperative screening tools could allow care teams to proactively allocate resources, such as social work, transportation assistance, or case management, for high-risk patients. Increasing wait times have been associated with increased mortality as well as heart failure rehospitalization [13]. Given the known association between prolonged time to TAVR and increased mortality [5,6], addressing these delays is not just a matter of equity but of clinical urgency.

This study has several limitations. First, while ADI is a validated metric, it may not capture individual-level barriers or the full nuance of patient-specific socioeconomic challenges. Second, we did not assess mortality or morbidity outcomes in this study, which limits the ability to draw conclusions about the clinical impact of observed delays. Additionally, this retrospective study focuses only on a single center. In order to determine if these trends are similar more globally, it would be prudent to examine other hospitals and cities.

## Conclusions

AVR is an increasingly popular form of aortic valve replacement. This study focuses on where improvements could be made to better access to TAVR and the consequential effects of such. We previously examined this dynamic in the context of the COVID-19 pandemic by comparing the time from first contact to TAVR across three pandemic waves at our institution relative to pre-COVID-19 data. COVID-19 increased our institution’s time to TAVR significantly across two waves, with an increase or decrease in all-cause mortality in each wave. This study highlights the importance of institutions developing mechanisms to ensure access to care during crises so that patients do not face potentially avoidable harm. In our cohort of 708 patients treated from 2019 to 2024, ADI was not associated with time from first structural heart team contact to implant for most patients when time to TAVR fell within the usual range. The signal showed up in the far right tail. Among patients with extreme delays, higher ADI and higher national ADI percentile were moderately associated with longer time to TAVR. In other words, deprivation did not shift the curve for everyone, but it seems to identify a subset most at risk of getting stuck in the workup pathway. This pattern is clinically plausible. Once a patient is in the TAVR pipeline, standardized protocols and multidisciplinary coordination may decrease some socioeconomic effects for routine cases. But when the process becomes non-routine, extra testing, missed connections, transportation issues, prior authorization delays, concurrent illness, or difficulty coordinating multiple appointments, background barriers can compound. Importantly, we did not find consistent evidence that the prolonged time in the outlier group was primarily explained by simple follow-up noncompliance. This suggests the delay is more likely driven by a mix of patient logistics and system-level friction rather than a single dominant cause.

Practically, ADI may be useful as an early flag for patients who need more support up front, including case management, social work, transport assistance, tighter scheduling of CT or catheterization, or dental clearance, and more proactive outreach, to prevent a small subset from developing extreme delays. Future work should validate these findings in multi-center cohorts, use analytic approaches that directly model the upper tail of time to TAVR, and link delay to outcomes in this population. From our data, the takeaway is straightforward. Neighborhood deprivation does not appear to drive routine time to TAVR at our institution, but it does track with the patients who experience the longest delays, and that is the group where targeted process fixes are most likely to matter.

## References

[1] Smith CR, Leon MB, Mack MJ (2011). Transcatheter versus surgical aortic-valve replacement in high-risk patients. N Engl J Med.

[2] Thourani VH, Kodali S, Makkar RR (2016). Transcatheter aortic valve replacement versus surgical valve replacement in intermediate-risk patients: a propensity score analysis. Lancet.

[3] Osnabrugge RL, Mylotte D, Head SJ (2013). Aortic stenosis in the elderly: disease prevalence and number of candidates for transcatheter aortic valve replacement: a meta-analysis and modeling study. J Am Coll Cardiol.

[4] Carroll JD, Mack MJ, Vemulapalli S (2021). STS-ACC TVT Registry of Transcatheter Aortic Valve Replacement. Ann Thorac Surg.

[5] Wijeysundera HC, Wong WW, Bennell MC, Fremes SE, Radhakrishnan S, Peterson M, Ko DT (2014). Impact of wait times on the effectiveness of transcatheter aortic valve replacement in severe aortic valve disease: a discrete event simulation model. Can J Cardiol.

[6] Billy MJ, Brennan Z, Ahmad T, Conte JV, Wallen TJ (2023). Time from first contact with heart team to transcatheter aortic valve replacement in the COVID-19 era. Cureus.

[7] Borkowski P, Borkowska N, Mangeshkar S, Adal BH, Singh N (2024). Racial and socioeconomic determinants of cardiovascular health: a comprehensive review. Cureus.

[8] Ram C, Yousef S, Ma WG (2024). Living in disadvantaged neighborhoods linked to less intervention for severe aortic stenosis. Sci Rep.

[9] Maroko AR, Doan TM, Arno PS, Hubel M, Yi S, Viola D (2016). Integrating social determinants of health with treatment and prevention: a new tool to assess local area deprivation. Prev Chronic Dis.

[10] (2025). University of Wisconsin School of Medicine and Public Health, Center for Health Disparities Research. About the Neighborhood Atlas® and area deprivation index (ADI). https://www.neighborhoodatlas.medicine.wisc.edu.

[11] David G, Bergman A, Gunnarsson C, Ryan M, Chikermane S, Thompson C, Clancy S (2024). Limited access to aortic valve procedures in socioeconomically disadvantaged areas. J Am Heart Assoc.

[12] Zaheer A, Qiu F, Manoragavan R, Madan M, Sud M, Mamas MA, Wijeysundera HC (2024). Impact of neighborhood social deprivation on delays to access for transcatheter aortic valve replacement: wait-times and clinical consequences. J Am Heart Assoc.

[13] Elbaz-Greener G, Masih S, Fang J (2018). Temporal trends and clinical consequences of wait times for transcatheter aortic valve replacement: a population-based study. Circulation.

